# Supplementary figures and images for: Specific and sensitive loop-mediated isothermal amplification (LAMP) method for Madurella strains, eumycetoma filamentous fungi causative agent
Source: PLoS Negl Trop Dis. 2023 Sep 18;17(9):e0011644. doi: 10.1371/journal.pntd.0011644 (PMC10538720; doi:10.1371/journal.pntd.0011644)

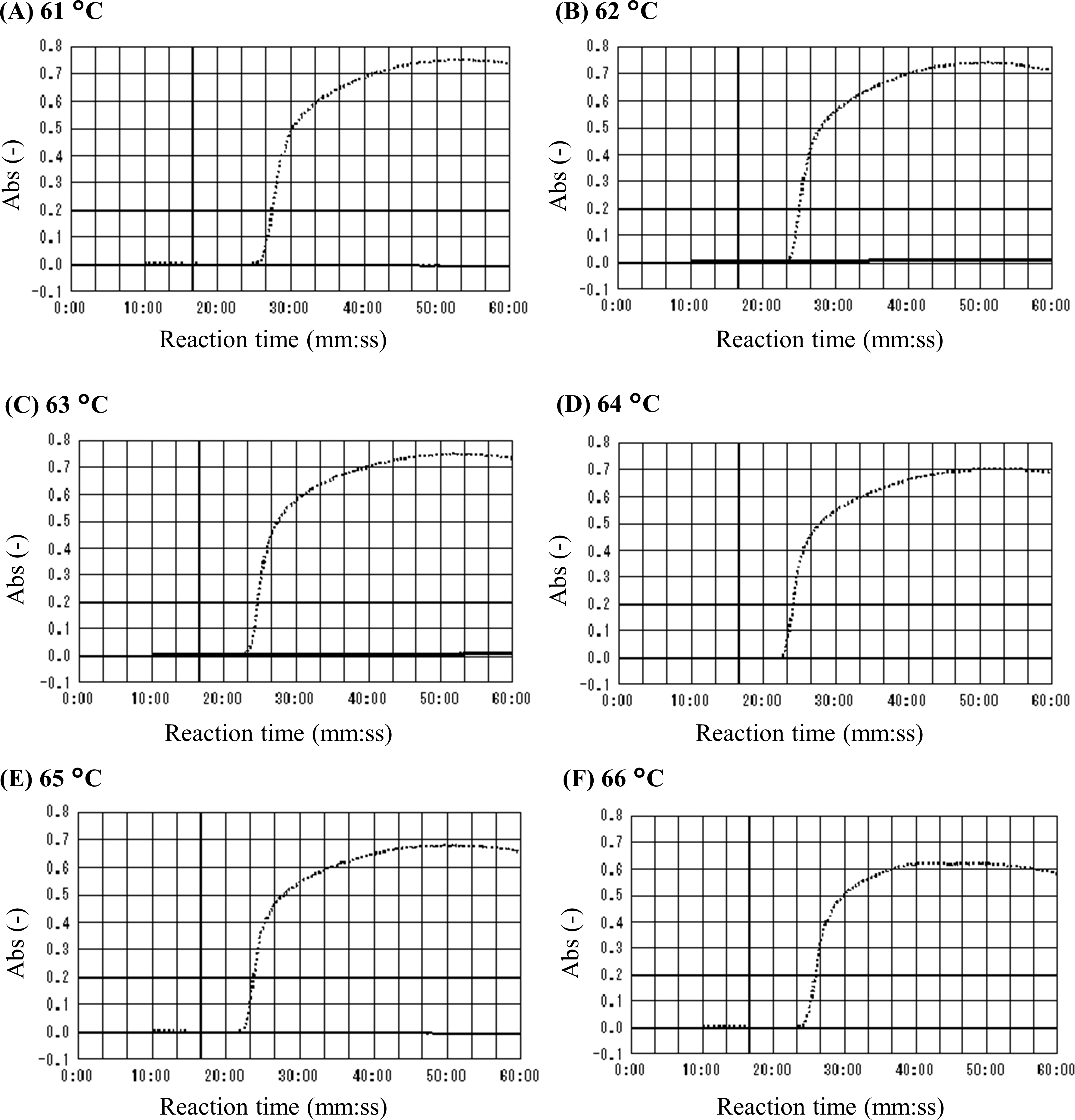

Supplement: S1 Fig — LAMP reaction was performed using genomic DNA of Madurella myceromatis IFM 46458 at the reaction temperature between 61°C and 66°C. The solid line and dotted line represent the data of the genomic DNA sample and blank using a buffer used for DNA dilution, respectively. (TIF) [file pntd.0011644.s001.tif]

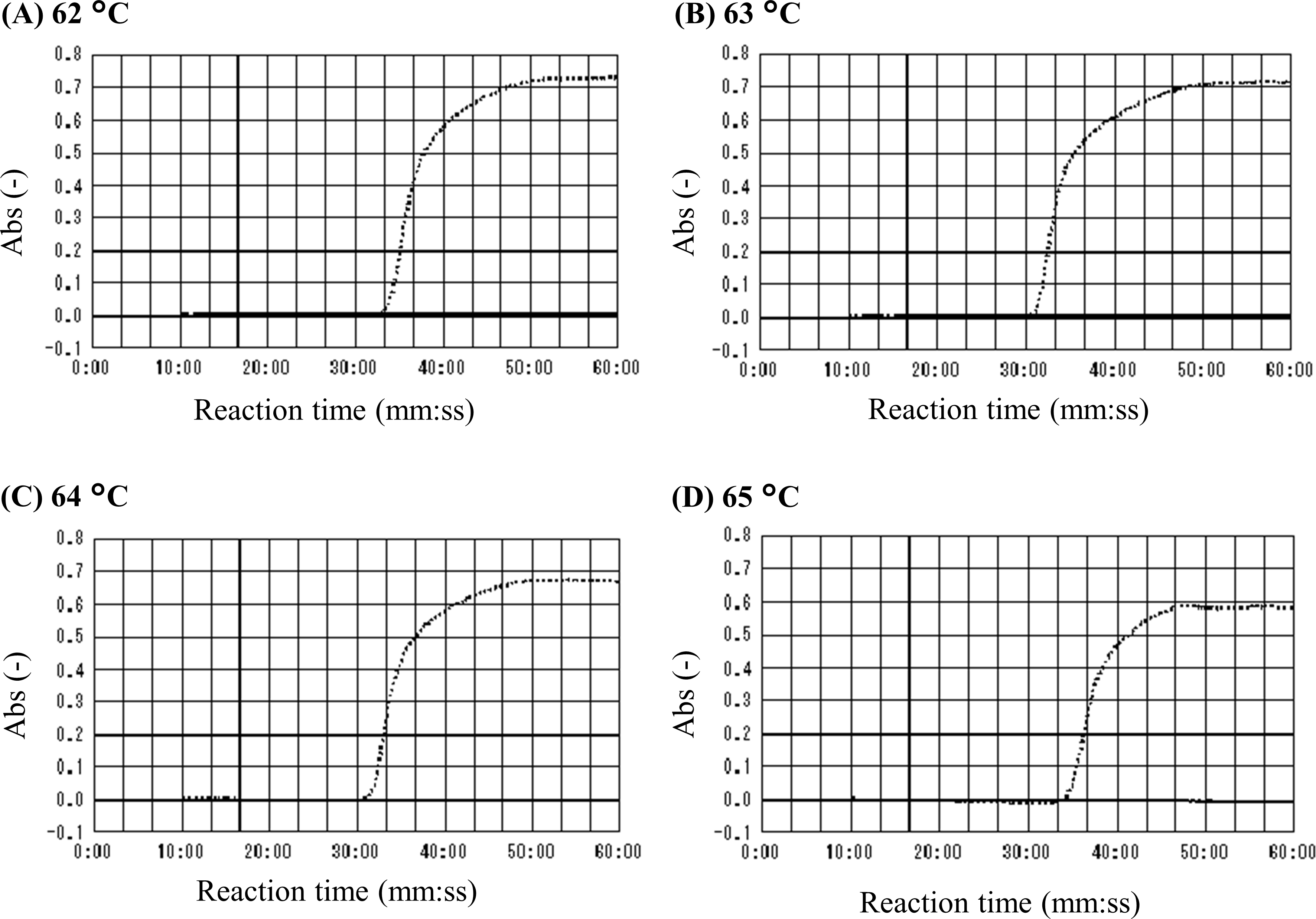

Supplement: S2 Fig — LAMP reaction was performed using genomic DNA of Madurella myceromatis IFM 46458 at the reaction temperature between 62°C and 65°C. The solid line and dotted line represent the data of the genomic DNA sample and blank using a buffer used for DNA dilution, respectively. (TIF) [file pntd.0011644.s002.tif]

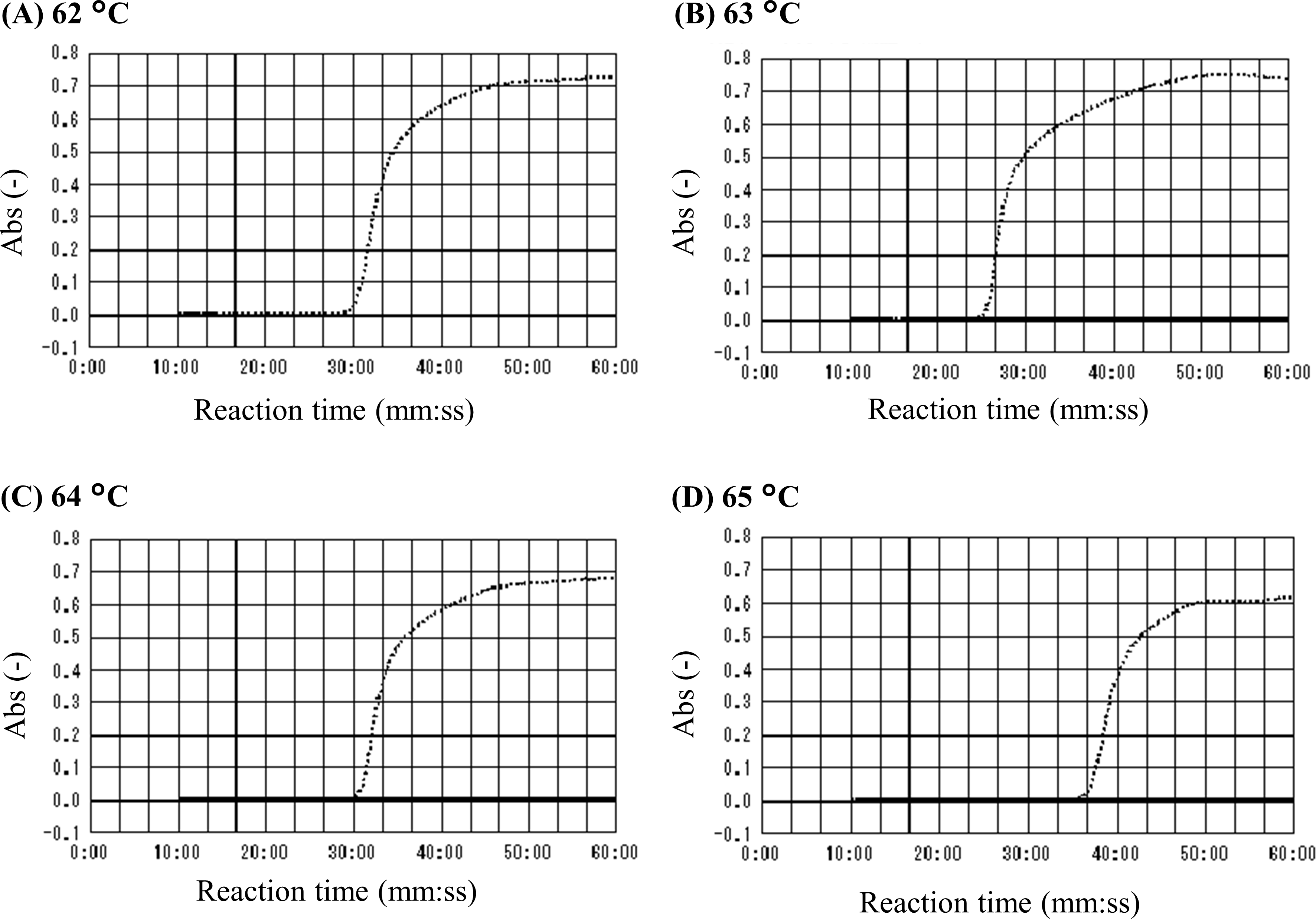

Supplement: S3 Fig — LAMP reaction was performed using genomic DNA of Madurella fahalii CBS 129176 at the reaction temperature between 62°C and 65°C. The solid line and dotted line represent the data of the genomic DNA sample and blank using a buffer used for DNA dilution, respectively. (TIF) [file pntd.0011644.s003.tif]

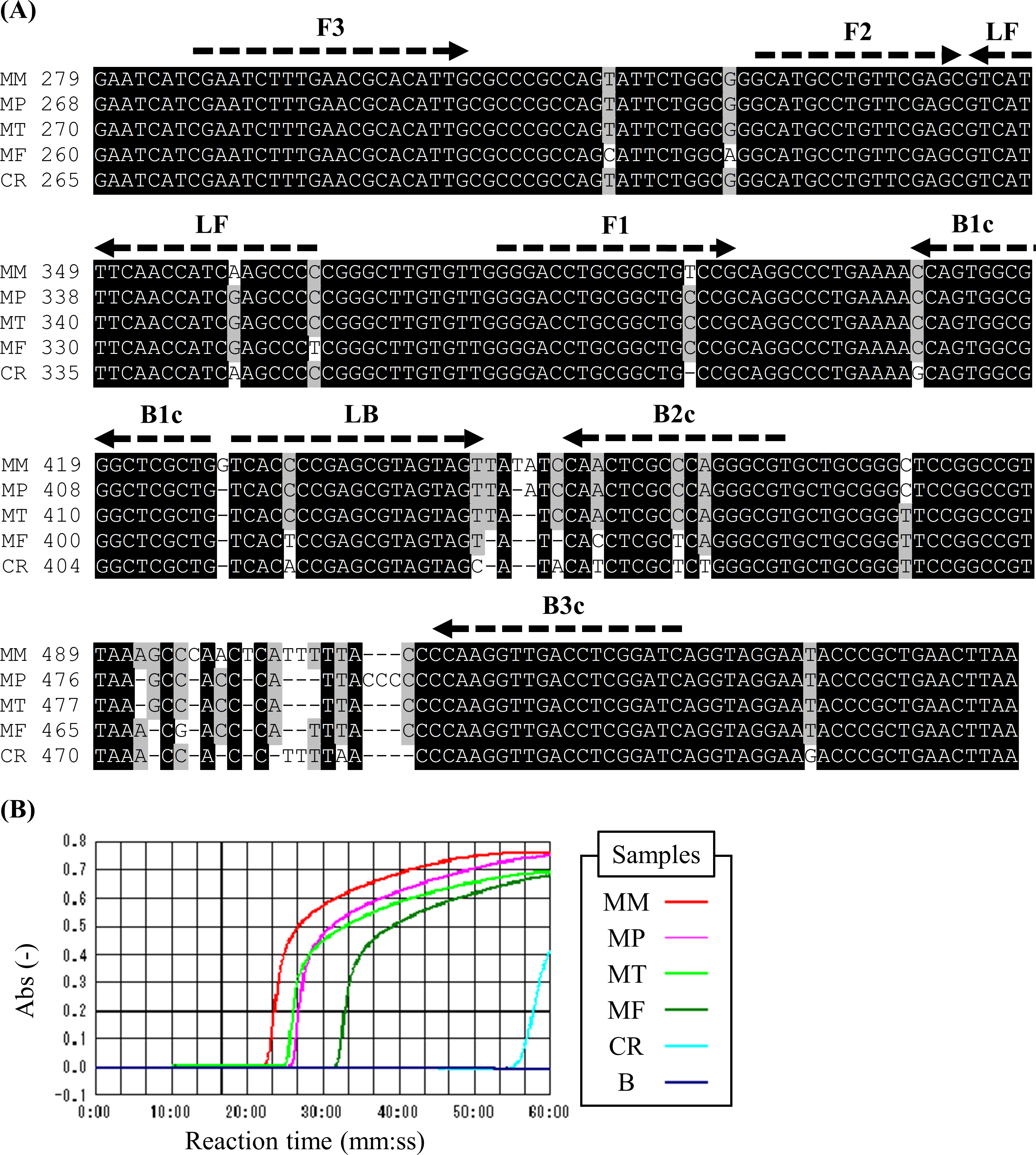

Supplement: S4 Fig — (A) The alignment of rDNA sequences among Madurella strains and Chaeromium rectangluare (negative control) is shown. Arrows illustrate the primers’ positions. (B) The specificity of primer PV2. LAMP reaction was performed using genomic DNAs derived from Madurella spp. and C. rectangulare at 65°C. Abbreviations: MM, M. mycetomatis; MP, M. pseudomycetomatis; MT, M. tropicana; MF, M. fahalii; CR, Chaetomium rectangluare; B, Blank sample with a buffer used for DNA dilution. (TIF) [file pntd.0011644.s004.tif]

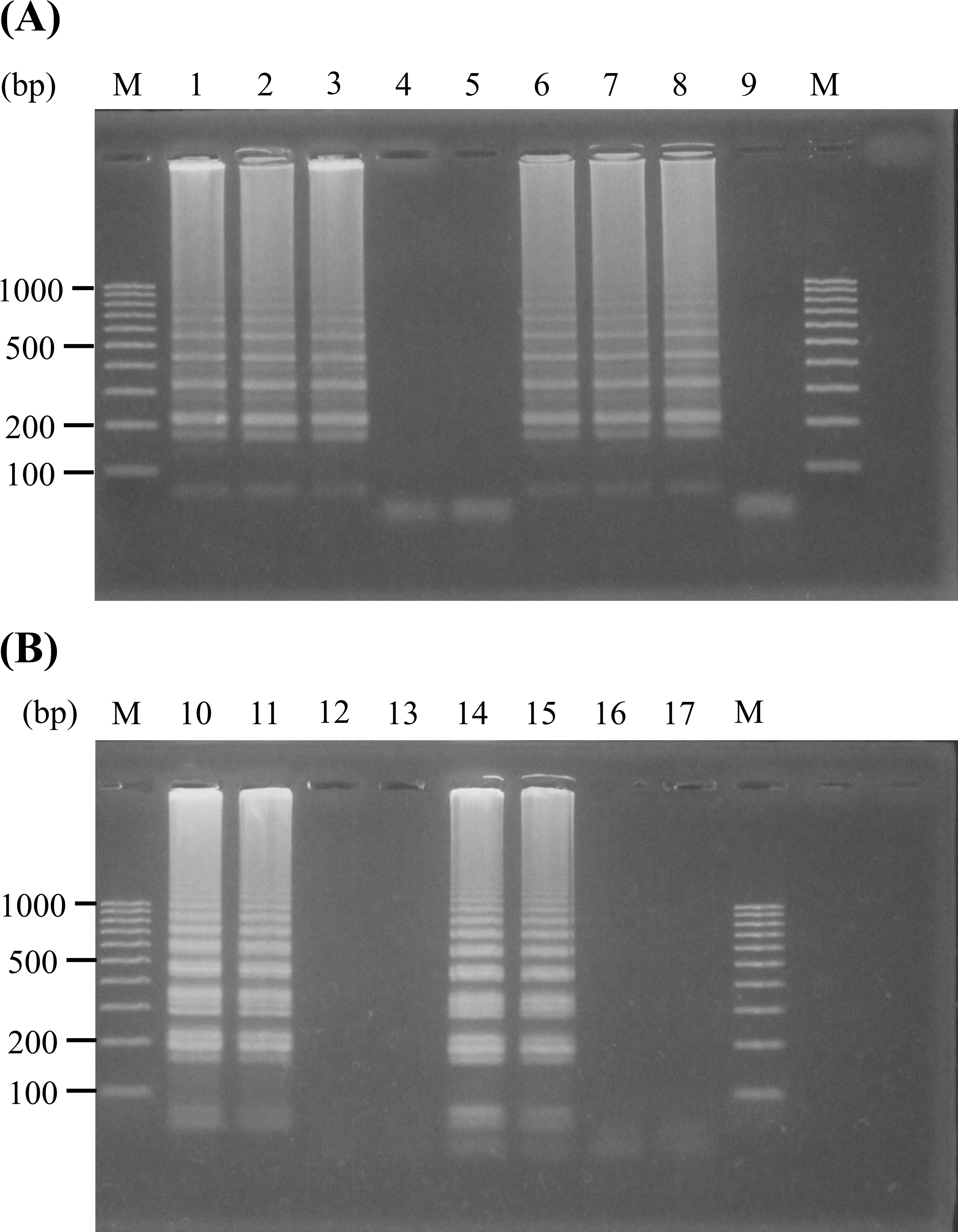

Supplement: S5 Fig — (A) PM (Lanes 1–9) and (B) PM (Lanes 10–13) and PF (Lanes 14–17) by agarose electrophoresis. Lanes: M, EZ load 100 bp Molecular Ruler (Bio-rad, Hercules, CA); 1, Madurella mycetomatis IFM 46458 (10 ng); 2, M. pseudomycetomatis IFM 46460 (10 ng); 3, M. tropicana CBS 201.38 (10 ng); 4, M. fahalii CBS 129176 (10 ng); 5, Chaetomium rectangulare CBS 126778 (10 ng); 6, M. mycetomatis IFM 46458 (1 pg); 7, M. pseudomycetomatis IFM 46460 (1 pg); 8, M. tropicana CBS 201.38 (1 pg); 9, no template; 10, M. mycetomatis IFM 46458 (10 ng); 11, M. mycetomatis IFM 46458 (1 pg); 12, M. fahalii CBS 129176 (10 ng); 13, no template; 14, M. fahalii CBS 129176 (10 ng); 15, M. fahalii CBS 129176 (1 pg); 16, M. mycetomatis IFM 46458 (10 ng); 17, no template. (TIF) [file pntd.0011644.s005.tif]
