# Supplementary material for: Specific and sensitive loop-mediated isothermal amplification (LAMP) method for Madurella strains, eumycetoma filamentous fungi causative agent
Source: PLoS Negl Trop Dis. 2023 Sep 18;17(9):e0011644. doi: 10.1371/journal.pntd.0011644 (PMC10538720; doi:10.1371/journal.pntd.0011644)
Supplement: Attachment — Submitted filename: Response_comment.docx [file pntd.0011644.s006.docx]

Reviewer 1

We thank you for your invaluable comments and suggestions. We have revised the manuscripts according to your comments. Hereafter, the revised manuscript is referred to as “2nd manuscript.” We highlighted the changes based on the reviewers’ comments using red letters, for your review.

**(Q1) My one recommendation would be that it would add to the manuscript to show the electrophoretic analyses of the results of the various LAMP protocols with the different organisms, rather than just the amplification curves. This would be important for centres who might wish to apply the protocol but who only have access to gel electrophoresis as end-point analysis.**

(A1)

We thank for your valuable suggestions. Following your comments, we performed agarose gel electrophoresis and added its results to 2nd manuscript as Supplementary Data (Fig. S5). In addition, the description of the experiments was added as followings:

**2nd Manuscript: Page 15, Lines 225-229**

Finally, the specificity of the LAMP reaction was verified using agarose gel electrophoresis. As shown in Fig. S5, the resultant solution in the positive samples showed the typical ladder bands while none of amplicons were detected in the reaction using non-target sample and no template. These results suggest that the expected amplification occurred.

**Other Reactions**

(R1)

In accordance with other reviewer’s comments, the title was changed. In brief, we added the description that this study did not use clinical specimens, and deleted the word “detect”. Additionally, the abstract and introduction section was slightly changed as followings:

**2nd Manuscript: Page 2, Lines 31-34 (Abstract)**

In this study, we developed the loop-mediated isothermal amplification (LAMP) method to present a foundational technique of the diagnosis of *Madurella* spp. (*M. mycetomatis*, *M. pseudomycetomatis*, *M. tropicana,* and *M. fahalii*), the common causative organisms of eumycetoma.

**2nd Manuscript: Page 18, Lines 247-250**

Since isothermal DNA amplification techniques are regarded as a promising tool in community-based medical treatment, this study aimed to develop a LAMP method for *Madurella* spp.

**2nd Manuscript: Page 19, Lines 282-286**

On the other hand, the data obtained from this study suggest that the LAMP method can also identify *Madurella* spp. using small amount of DNA (>1 pg) at the endpoint in one batch. Thus, the LAMP method might be an alternative to PCR as a diagnostic method for mycetoma in community-based medicine.

(R2)

In accordance with other reviewer’s comments, the limitation of this study was also discussed as followings:

**2nd Manuscript: Page 18, Lines 264-267**

On the other hand, *M. pseudomycetomatis* and *M. tropicana* have only been tested in single strains each. Thus, a larger number of strains belonging to these species should be tested in the future to verify the specificity of these primers.

**2nd Manuscript: Page 19-20, Lines 290-295**

… In addition, the preparation of crude DNA derived from mycetoma specimens for PCR diagnosis has been reported [13,16]. These findings support the idea that our LAMP system will be applicable to clinical samples. However, in this study, specimens of mycetoma were not used and the LAMP reaction was performed only in the laboratory. Thus, the further study in clinical sites is required for practical use of this technique.

(R3)

We added the explanation of Fig. S4, result of other candidate primer available for multiple *Madurella* spp. (primer set PV2). We are sincerely sorry that the data has been missed.

**2nd Manuscript: Page 14-15, Lines 212-218**

On the other hands, we also designed the primer pair (primer set PV2) based on the ITS-2 region, which has potential to amplify all four *Madurella* spp. due to its highly conserved sequence, as shown in Fig. S4. As expected, primer PV2 could amplify the genomic DNAs of four *Madurella* spp., but it also showed the reactivity to *C. rectangulare*, at an optimal reaction temperature (65°C). Thus, we performed further experiments using the primers PV, PM and PF as basal primer sets.

**2nd Manuscript: Page 18, Lines 260-264**

Similarly, the double check for *M. fahalii* will become significant because it is known to exhibit azole-resistance. To overcome this problem, the primer set targeting all *Madurella* spp. is needed. Unfortunately, such primer sets were not obtained as far as the rDNA ITS regions were used as target, as shown in Fig. 4A and Fig. S4.

(R4)

In accordance with other reviewer’s comments, the number of isolates to test LAMP primer sets increased. Thus, Tables 2 and 3 has been changed and the descriptions concerning this revision have been added into 2nd manuscript as followings:

**2nd Manuscript: Page 5, Lines 105-117**

A total of 34 fungal- and 5 bacterial strains were used in this study (Table 1). The details of fungal strains are as follows: 16 belong to *Madurella*, three to other members of *Chaetomiaceae*, four to *Aspergillus,* and 11 to other taxa recognized as causative agents of mycetoma [3,11]. The species of five bacterial strains used in this study are also known to cause actinomycetoma [2]. Briefly, 14 clinical strains were preserved at Medical Mycology Research Center (MMRC; Chiba University, Japan) as IFM collection through the National Bio-Resource Project, Japan, and 13 mycetoma strains preserved at the Mycetoma Research Center (MRC) Khartoum University, Khartoum, Sudan. In addition, 12 strains were obtained from the culture collections of the Fungal Biodiversity Center (CBS; Netherlands), NITE Biological Resource Center (NBRC; Japan), American Type Culture Collection (ATCC; United States) and Japan Collection of Microorganisms (JCM; Japan).

**2nd Manuscript: Page 9, Lines 126-132**

**All strains were grown on a PDA medium at 25°C for 1–3 weeks. Bacterial colonies or mycelia were picked by inoculating the needle and suspended in 500 μL of 100 mM Tris-HCl buffer (pH 8.5) containing 40 mM EDTA. The bacterial cell or fungus body in the suspension was disrupted using MagNA Lyser and MagNA Lyser Green Beads (Roche Diagnostics, Indianapolis, IN) at 5000 rpm for 20 s. Genomic DNAs were extracted using the benzyl chloride method [23] with some modifications.**

Reviewer 2

We thank you for your invaluable comments and suggestions. We have revised the manuscripts according to your comments. Hereafter, the revised manuscript is referred to as “2nd manuscript.” We highlighted the changes based on the reviewers’ comments using red letters, for your review.

**(Q1) However, the value of this test method is limited if it cannot be implemented in Eumycetoma endemic areas; currently, the gene amplification system is only being executed in Japan. Field validation tests are urgently needed. Additionally, the number of positive and negative control fungal species used seems insufficient. Conversely, the descriptions about the amplification system are overly general and verbose, requiring substantial reduction.**

(A1-1)

We thank for your suggestion. As you pointed out, it is important to mention the limitation of this study. Thus, we added the descriptions that a larger number of *Madurella* strains are needed for validation and that this study was performed only in the laboratory, as followings:

**2nd Manuscript: Page 18, Lines 264-267**

On the other hand, *M. pseudomycetomatis* and *M. tropicana* have only been tested in single strains each. Thus, a larger number of strains belonging to these species should be tested in the future to verify the specificity of these primers.

**2nd Manuscript: Page 19-20, Lines 290-295**

… In addition, the preparation of crude DNA derived from mycetoma specimens for PCR diagnosis has been reported [13,16]. These findings support the idea that our LAMP system will be applicable to clinical samples. However, in this study, specimens of mycetoma were not used and the LAMP reaction was performed only in the laboratory. Thus, the further study in clinical sites is required for practical use of this technique.

(A1-2)

In addition, some explanations were added in order to avoid misunderstandings and to highlight the point that this study developed a fundamental LAMP system for detection of *Madurella* in the laboratory while it does not refer to field experiments.

**2nd Manuscript: Page 2, Lines 31-34 (Abstract)**

In this study, we developed the loop-mediated isothermal amplification (LAMP) method to present a foundational technique of the diagnosis of *Madurella* spp. (*M. mycetomatis*, *M. pseudomycetomatis*, *M. tropicana,* and *M. fahalii*), the common causative organisms of eumycetoma.

**2nd Manuscript: Page 18, Lines 244-249**

Since isothermal DNA amplification techniques are regarded as a promising tool in community-based medical treatment, this study aimed to develop a LAMP method for *Madurella* spp.

**2nd Manuscript: Page 19, Lines 282-286**

On the other hand, the data obtained from this study suggest that the LAMP method can also identify *Madurella* spp. using small amount of DNA (>1 pg) at the endpoint in one batch. Thus, the LAMP method might be an alternative to PCR as a diagnostic method for mycetoma in community-based medicine.

**(Q2) Title: The title reads, "Specific and Sensitive loop-mediated isothermal amplification (LAMP) detection method for Madurella strains, a eumycetoma filamentous fungi causative agent." However, this paper does not detect from clinical or environmental samples but merely identifies from fungal strain DNA using LAMP. The word "detection" may cause confusion and should be removed. A more concise alternative could be: "Specific and Sensitive LAMP method for Madurella strains, a eumycetoma causative agent."**

(A2)

The title was revised by following your suggestion.

**(Q3) Abstract: p.2 L. 28 mentions "Sudan." Eumycetoma is not exclusive to Sudan, and given that the research discussed in this paper isn't region-specific, the wording should be adjusted. Similar changes are needed in the Introduction.**

(A3)

Thank you for your suggestions. We changed the sentences to highlight the mycetoma-endemic region, as followings:

**2nd Manuscript: Page 2, Lines 27-28 (Abstract)**

Filamentous fungi of the genus *Madurella* are the primary causative agents of mycetoma, a disease observed in tropical and subtropical regions.

**2nd Manuscript: Page 3, Lines 68-69**

It is endemic to tropical and subtropical areas (the mycetoma belt) [1,2]. Especially, eumycetoma has been frequently reported in African countries [2,3].

**(Q4) Abstract: p.2 L. 32, “(LAMP) detection” should be modified to avoid misunderstanding as the paper validates identification but doesn't discuss detection from various samples. Thus, "detection" should be removed.**

(A4)

Following your suggestions, we deleted the term “detection” through the manuscript. However, to discuss the efficiency of LAMP method, “detect/detection” might be an essential word and not lead to misunderstanding. For example, “detection limits”. Thus, we partially remained the word in the sentence, where it is obvious that the validation of LAMP method had been performed. We would be grateful if you could understand our intent.

**(Q5) Abstract: p.3 L. 57, "to detect Madurella strains" should be changed to "to identify Madurella strains."**

(A5)

The corresponding part was revised by following your suggestion.

**(Q6) Introduction: p.3 L.68, a reference is required for "(the mycetoma belt)."**

(A6)

Thank you for your suggestions. We have added the reference to describe the mycetoma belt.

**(Q7) Fungal Strains: p.5, The number of strains for both Positive and Negative controls is too few. It's acceptable for Madurella mycetomatis with 10 strains and Madurella fahalii with just 4 strains. However, including species like Madurella pseudomycetomatis and Madurella tropicana, which are only studied for one strain, is questionable. The paper should clearly state these limitations. Moreover, many known Eumycetoma causative agents like Falciformispora senegalensis, Curvularia lunata, Scedosporium spp., Zopfia rosatii, Acremonium spp., and Fusarium spp. are listed in the WHO FPPL. The study should consider including them. For differentiation, it should also look into the causative agents of Actinomycetoma.**

(A7)

This indication is an significant point. First, regarding the number of *Madurella* strains, we revise the discussion in 2nd manuscript as described in (A1). Second, as for the other mycetoma-causative agents, we performed further experiments and added the results into 2nd manuscript as written below. In addition, Tables 2 and 3 were also revised. Thank you for your valuable suggestions.

**2nd Manuscript: Page 5, Lines 105-117**

A total of 34 fungal- and 5 bacterial strains were used in this study (Table 1). The details of fungal strains are as follows: 16 belong to *Madurella*, three to other members of *Chaetomiaceae*, four to *Aspergillus,* and 11 to other taxa recognized as causative agents of mycetoma [3,11]. The species of five bacterial strains used in this study are also known to cause actinomycetoma [2]. Briefly, 15 clinical strains were preserved at Medical Mycology Research Center (MMRC; Chiba University, Japan) as IFM collection through the National Bio-Resource Project, Japan, and 13 mycetoma strains preserved at the Mycetoma Research Center (MRC) Khartoum University, Khartoum, Sudan. In addition, 12 strains were obtained from the culture collections of the Fungal Biodiversity Center (CBS; Netherlands), NITE Biological Resource Center (NBRC; Japan), American Type Culture Collection (ATCC; United States) and Japan Collection of Microorganisms (JCM; Japan).

**2nd Manuscript: Page 9, Lines 126-132**

**All strains were grown on a PDA medium at 25°C for 1–3 weeks. Bacterial colonies or mycelia were picked by inoculating the needle and suspended in 500 μL of 100 mM Tris-HCl buffer (pH 8.5) containing 40 mM EDTA. The bacterial cell or fungus body in the suspension was disrupted using MagNA Lyser and MagNA Lyser Green Beads (Roche Diagnostics, Indianapolis, IN) at 5000 rpm for 20 s. Genomic DNAs were extracted using the benzyl chloride method [23] with some modifications.**

**(Q8) Table 1: p.8, “Chatomium lectangulare” should be corrected to “Chaetomium lectangulare.”**

(A8)

The corresponding part was revised by following your suggestion.

**(Q9) Preparation of fungal genomic DNA: p.8, The DNA quantification method is not mentioned, which is crucial for evaluating LAMP detection sensitivity.**

(A9)

As you pointed out, this is an important information. Thus, we added the description into 2nd manuscript as followings:

**2nd Manuscript: Page 9-10, Lines 132-135**

Finally, the concentration of DNA solution was determined by absorbance at 260 nm using microvolume spectrophotometer DS-11 (Denovix, Wilmington, DE) and the solution was stored at 4°C in Tris-EDTA buffer.

**(Q10) Table 2 & Fig S4: p. 10 & 35-36, No mention of the PV2 primer set is found in the main text. Based on Fig S4, its specificity is questionable as the Negative Control shows a reaction within 60 minutes. Both should be removed.**

(A10)

We are sincerely sorry that the explanation of primer PV2. However, Fig. S4 is an important result to support the idea that only the primer set PV is available for detection of multiple *Madurella* spp. while ITS2 region seems to be well-conserved. Thus, this discussion was also revised as followings:

**2nd Manuscript: Page 14-15, Lines 212-218**

On the other hands, we also designed the primer pair (primer set PV2) based on the ITS-2 region, which has potential to amplify all four *Madurella* spp. due to its highly conserved sequence, as shown in Fig. S4. As expected, primer PV2 could amplify the genomic DNAs of four *Madurella* spp., but it also showed the reactivity to *C. rectangulare*, at an optimal reaction temperature (65°C). Thus, we performed further experiments using the primers PV, PM and PF as basal primer sets.

**2nd Manuscript: Page 18, Lines 260-264**

Similarly, the double check for *M. fahalii* will become significant because it is known to exhibit azole-resistance. To overcome this problem, the primer set targeting all *Madurella* spp. is needed. Unfortunately, such primer sets were not obtained as far as the rDNA ITS regions were used as target, as shown in Fig. 4A and Fig. S4.

**(Q11) Design of the primer pairs detecting Madurella spp.: p.12, While Chaetomium spp. is known as a causative agent of Eumycetoma, no evidence of Eumycetoma caused by Chaetomium lectangulare was found. Is it really a causative agent or just used due to sequence similarity? An explanation is needed.**

(A11)

As you mentioned, *Chaetomium rectangulare* was chosen only based on sequence similarity. Thus the description was added into 2nd manuscript.

**2nd Manuscript: Page 13, Lines 176-181**

A negative control strain was searched using NCBI BLAST with the rDNA sequence of *M.* *mycetomatis* as the query, and *Chaetomium rectangulare* (accession no. NR_144817) showed the highest similarity among the strains preserved in the MRC. Therefore, *C. rectangulare* CBS 126778 was selected as the negative control to validate specificity, though there is no report that it causes mycetoma.

**(Q12) Validation of LAMP primers: p.12-13, The discussion here is merely about basic reaction conditions. This section should be simplified, and some of the figures could be removed.**

(A12)

One of the most important points in this study is that our LAMP method can identify the *Madurella* spp. at the same reaction temperature (= in one batch reaction). Thus, the proof of the optimal reaction temperature is significant. In addition, the detection limits of primer sets might be important information. We would be grateful if you could understand our intent.

**(Q13) LAMP amplification using clinical isolates and other genera: p.13-14, The subtitle "and other genera" seems redundant. Additionally, on p.14 L 221, does "clinical samples" actually mean "clinical isolates"?**

(A13)

As you pointed out, “and other genera” seems redundant. However, some of strains used in this study did not originate from mycetoma specimens but they belong to species known as causative agents. Thus, to avoid misunderstanding, the header of this section was changed to “LAMP amplification using known causative species”, and a description was added.

**2nd Manuscript: Page 15, Lines 232-234**

To verify the availability of the designed primers, we performed a LAMP reaction using 10 ng DNA from mycetoma clinical isolates and species known as the mycetoma causative agents including bacteria [3,11,16].

**(Q14) Table 3: p.14-15, The letter "N" is used in the table, but there's no explanation in the footnotes. If it means "not tested," considering the common fungal species, shouldn't they be tested?**

(A14)

The letter “N” means “not tested”, as you mentioned. Because if the result of primer sets PV and PF showed negative, the species of sample should be excluded from *Madurella*, as shown Table 3. However, you question is reasonable. Thus, we performed further experiments and showed new results in Table 3.

**(Q15) Discussion: p15-18, This section is lengthy and should be compressed by at least 66%.**

(A15)

Thank you for your valuable suggestions for improvement of our manuscript. Following your suggestions, we reduced the sentences in the Discussion Section. However, some of new discussion was added in the process of revision process. Please check the revised Discussion Section.

**Other Reactions**

(R1)

In accordance with another reviewer’s comments, we performed agarose gel electrophoresis and added its results to 2nd manuscript as Supplementary Data (Fig. S5). In addition, the description of the experiments was added as followings:

**2nd Manuscript: Page 15, Lines 225-229**

Finally, the specificity of the LAMP reaction was verified using agarose gel electrophoresis. As shown in Fig. S5, the resultant solution in the positive samples showed the typical ladder bands while none of amplicons were detected in the reaction using non-target sample and no template. These results suggest that the expected amplification occurred.
